# Subacute Testicular Toxicity to Cadmium Exposure Intraperitoneally and Orally

**DOI:** 10.1155/2019/3429635

**Published:** 2019-11-25

**Authors:** Viviane G. S. Mouro, Ana L. P. Martins, Janaina Silva, Tatiana P. Menezes, Marcos L. M. Gomes, Juraci A. Oliveira, Fabiana C. S. A. Melo, Sérgio L. P. Matta

**Affiliations:** ^1^Department of General Biology, Federal University of Viçosa, Viçosa, MG 36570-900, Brazil; ^2^Department of Structural Biology, Federal University of Triângulo Mineiro, Uberaba, MG 38025-180, Brazil; ^3^Department of Animal Biology, Federal University of Viçosa, Viçosa, 36570-900 MG, Brazil

## Abstract

The toxic effects of cadmium (Cd) on reproductive parameters are widely described in the literature. Experimental models often make use of the intraperitoneal route (*i.p.*), although human intoxication occurs preferentially by the oral route and can be continuous. However, little is known about the effect of Cd administration routes on the testicular structure. Thus, this study investigated the testicular impact of Cd exposure comparing both *i.p.* and oral routes, both single dose (SD), in addition to the oral route in fractional doses (FD). Swiss adult male mice received CdCl_2_ 1.5 mg/kg *i.p.*, 30 mg/kg oral SD, and 4.28 mg/kg oral FD for 7 consecutive days. The Cd bioaccumulation was observed in all routes, mainly in the oral FD route. The concentrations of testicular Ca and Cu decreased in all animals exposed to Cd, while Zn and Mn decreased only in the *i.p.* route. Testicular SOD activity was reduced in both routes of oral administration, while CAT increased in the *i.p.* route, and GST increased in all animals exposed to Cd. Changes in the tubular parameters and cell viability were observed in both routes of Cd administration but were more intense in the oral route, mainly in the FD. Serum testosterone concentration was reduced in both routes of oral administration. Tubular damage, such as the vacuolization of the seminiferous epithelium, germ cell detachment, and seminiferous tubule degeneration, occurred in all groups exposed to Cd. Therefore, the oral Cd administration presented greater potential to promote testicular damage, mainly when the metal was given in a fractionated way.

## 1. Introduction

Cadmium (Cd) is a nonessential metal that can contaminate by occupational and nonoccupational exposure. Occupational exposure is associated with paint, plastic, glass, metal alloy production, and mining activities [1]. Nonoccupational exposure can occur by the ingestion of food and water containing the metal and bioaccumulation in plants and aquatic organisms [2]. Other forms of contamination are inhalation of cigarette smoke or air pollution caused by forest fires, mining areas, and metal refining industries [3].

It is known that Cd exposure can affect organs, such as the liver, lung, and kidneys, and the testicles are particularly sensitive to toxicity mediated by this pollutant [4, 5]. Changes in testicular structure due to Cd intoxication include damage to germ and Sertoli cells, as well as degeneration and testicular necrosis [4, 6, 7], since this metal leads to the rupture of the blood-testis barrier [8, 9].

When ingested, Cd is absorbed in the duodenum by the divalent metal receptor (DMT-1), which absorbs microminerals [10, 11]. This competition may reduce the concentration of essential minerals, such as magnesium (Mg), iron (Fe), zinc (Zn), selenium (Se), and copper (Cu), which are important for the development and maintenance of spermatogenesis [6]. The aforementioned minerals act as cofactors of antioxidant enzymes, such as superoxide dismutase [12], and a reduction of mineral levels increases reactive oxygen species (ROS) concentrations, which is also a mechanism of Cd toxicity.

Matovic et al. [13] have shown that, when Cd is administered through intraperitoneal injection (*i.p.*) (1.5 mg/kg) and at the corresponding oral dose (30 mg/kg), both in single doses, a severe acute hepatic intoxication is observed in the *i.p*. route. However, Mouro et al. [14] found that testicular damage after Cd exposure was more intense in the oral single dose (24 mg/kg) compared to the *i.p.* (1.2 mg/kg) route in the subchronic evaluation. The authors did not observe histomorphometric changes in the testicular tissue, at the used doses, in the subacute evaluation. However, it is still unknown whether the dose, route, and frequency of administration (*i.p.* or oral, single or fractioned doses) cause similar testicular damage. Therefore, the present study is aimed at verifying if Cd exposure, by oral and *i.p.* single dose and fractionated oral route, may lead to the same changes in testicular reproductive parameters in adult mice in the subacute evaluation after Cd exposure.

## 2. Material and Methods

### 2.1. Animal Model

Twenty Swiss adult male mice (8 weeks old) were maintained under controlled illumination (12-12 h light/dark) and ambient temperature (21 ± 1°C). Water and feed, standard rodent diet, were offered *ad libitum*. The experimental procedures were approved by the Ethics Committee on Animal Use of the Federal University of Viçosa (protocol 058/2016) and were performed in accordance with the guidelines issued by the National Council for the Control of Animal Experimentation (CONCEA).

### 2.2. Experimental Design and Tissue Collection

After 1 month of adaptation, the mice were randomized in four experimental groups (*n* = 5 animals/group): one control group and three groups exposed to Cd. The control group received distilled water by gavage, while the other groups received a solution of cadmium chloride (CdCl_2_, Sigma, St Louis, MO, USA) at the following concentrations: 1.5 mg/kg (0.92 mg Cd/kg) *i.p.* single dose (*i.p.* SD), 30 mg/kg (18.33 mg Cd/kg) orally by gavage single dose (oral SD), and 4.29 mg/kg/day (2.62 mg Cd/kg/day) orally by gavage for seven consecutive days (fractionated dose, oral FD). After one week, both groups exposed to oral Cd received a total of 30 mg/kg CdCl_2_. The dose of 1.5 mg/kg of CdCl_2_ was chosen based on a previous study carried out by our laboratory (unpublished data). The oral dose of 30 mg/kg was chosen because Cd absorption into duodenal cells is approximately 5% of that which was orally ingested [15–17]. Thus, this dose corresponds to 1.5 mg/kg *i.p.* Furthermore, this amount is considered a safe standard rate of absorption after Cd exposure [3].

The 7-day period was adopted to observe subacute effects induced by Cd exposition before a possible rehabilitation process could change the degree of damage [4]. On the 8th day of the experiment, the animals were anesthetized (sodium thiopental 30 mg/kg *i.p.*) and euthanized by deepening the anesthesia (sodium thiopental, 150 mg/kg *i.p.*) followed by cardiac puncture and exsanguination. The testes were collected, dissected, and weighed.

The left testis of each animal was immersed in Karnovsky fixative solution, in which approximately half of each testis was destined for histological evaluation and cell viability whereas the other half was used to determine the dosage of Cd and microminerals. The right testis of each animal was frozen (-80°C) for assessment of oxidative and nitrosative stress markers.

### 2.3. Cadmium Bioaccumulation and Micromineral Levels

Samples of the testis designated to this were weighed in an analytical balance (0.0001 g; BEL Mark 210A) and dried (70°C), until the dry weight was constant. The dried samples were placed in Erlenmeyer flasks with 1.5 mL of concentrated nitric acid (HNO_3_) and 0.5 mL of perchloric acid (HClO_4_, 70%). Afterward, they were transferred to the hot plate. The temperature was gradually raised to 90°C so that the samples had a complete digestion. Then, the samples were diluted in deionized water in a 10 mL volumetric flask and filtered using filter papers. The concentrations of cadmium (Cd), zinc (Zn), calcium (Ca), magnesium (Mn), manganese (Mg), copper (Cu), and iron (Fe) were determined using an atomic absorption spectrophotometer (SpectrAA 220FS Varian) [14].

### 2.4. Assessment of Oxidative and Nitrosative Stress Markers

The frozen testes were homogenized with potassium phosphate buffer (pH 7.4, 0.1 M) containing 1 M EDTA, in the proportion of 100 mg of tissue for 1 mL of buffer, followed by centrifugation at 3000 g (6200 rpm) for 10 min. The analyses were performed in the supernatant and in duplicate.

The superoxide dismutase (SOD) activity was determined by the pyrogallol method, which is based on the ability of SOD to catalyze the superoxide radical (O^−2^) reaction in hydrogen peroxide (H_2_O_2_), monitored at 570 nm in a microplate spectrophotometer (PowerWave X) [18]. The catalase (CAT) activity was assayed according to Dieterich et al. [19], by measuring the decomposition rate of H_2_O_2_ for 60 seconds, in a spectrophotometer, at 240 nm. The glutathione S-transferase (GST) activity was estimated spectrophotometrically, at 340 nm [20], and calculated through the formation of 1-chloro-2,4-dinitrobenzene (CDNB) conjugate.

For determining MDA levels, the samples were placed to react with thiobarbituric acid reactive substance (TBARS) solution (15% trichloroacetic acid, 0.375% thiobarbituric acid, and HCl 0.25 N) for 40 min in a water bath (90°C). TBARS formation was monitored at 535 nm in a microplate spectrophotometer (PowerWave X) [21]. Tissue levels of NO were indirectly determined by the quantification of nitrite/nitrate levels through of the standard Griess reaction [22]. Briefly, the testicular samples were placed with an equal volume of Griess reagent (1% sulfanilamide, 0.1% naphthylethylenediamine hydrochloride, and 2.5% H_3_PO_4_), at room temperature, for 10 min. The absorbance was measured spectrophotometrically at 570 nm. The nitrite concentration was calculated with reference to the standard curve (0.0610 to 125 *μ*M) of sodium nitrite (NaNO_2_).

### 2.5. Histological Preparation

The left testis was immersed in Karnovsky fixative solution (4% paraformaldehyde: 4% glutaraldehyde in phosphate buffer 0.1 mol L^−1^, pH 7.4) [23] for 24 hours. The samples were dehydrated in crescent ethanol series and embedded in glycol methacrylate (Historesin, Leica Microsystems, Nussloch, Germany). Semiserial sections (3 *μ*m) were obtained using a rotatory microtome (RM2255, Leica Biosystems, Nussloch, Germany), with at list 40 *μ*m between sections. Then, the histological slides were stained with toluidine blue/sodium borate (1%). The digital images were obtained using a light-field photomicroscope (Olympus BX-53, Tokyo, Japan) connected to a digital camera (Olympus DP73, Tokyo, Japan). Finally, the histomorphometric assessment was performed using ImageJ® (National Institute of Health, USA) software.

### 2.6. Testicular Histopathology

For the histopathological evaluation of the testis, the proportion of normal and pathological seminiferous tubules was estimated by counting 200 random tubules per animal. The pathological tubules were separated as follows: mild pathologies (vacuolization and germinal epithelial detachment) and severe pathologies (absence of germ cells and/or Sertoli cells). This procedure was adapted from the Johnsen index [24].

### 2.7. Testicular Cell Viability

Histological slides containing testicular sections of 1 *μ*m were stained with acridine orange (AO; green) and propidium iodide (PI; red) for evaluating cellular morphological changes [25]. AO is a vital dye that stains both live (viable) and dead (nonviable) cells, while PI stain only stains cells that have lost their membrane integrity. Therefore, the cellular classification was based on nuclear condensation and fragmentation as well as membrane integrity [25].

Viable cells stained by AO exhibit intact green nuclei, while nonviable cells costained by AO and PI show a dense yellow/orange/red nucleus due to chromatin condensation and the degree of loss membrane integrity [26]. Digital images were obtained using the EVOS FL photomicroscope (Life Technologies, Carlsbad, Canada) and evaluated using ImageJ® image analysis software (Media Cybernetics, Silver Spring, MD). A total area of 30 × 10^4^ *μ*m^2^ per testis was used to calculate the percentage of nonviable germ cells within the seminiferous tubules [27].

### 2.8. Histomorphometric Evaluation of Seminiferous Tubules

The gonadosomatic index (GSI) was determined according to Amann [28] using the following equation: GSI = TW/BW × 100, in which TW is the testes weight and BW is the body weight. The weight of the testicular parenchyma (PW) was considered the weight of the testis without albuginea. The parenchymosomatic index (PSI) was calculated as follows: PSI = TW/BW × 100.

The proportion among the components that constitute the testicular parenchyma was obtained using square grids placed over digital images (100x magnification). A total of 2,660 intersection points were counted per animal classifying those that were on seminiferous tubules and their components (tunica propria, seminiferous epithelium, and lumen) and interstitial tissue. The percentage of each component was determined by the following equation: volume density (%) = (number of points in the tubule or intertubule/2,660 points in total) × 100. The volume (mL) of each component was obtained by the following equation: (%tubule or intertubule × testicular parenchyma weight)/100 [29]. Considering that the density of the mammalian testis is about 1 [30], the mass of the testicle is equal to its volume.

The tubulesomatic (TSI) and epithelium somatic (ESI) indexes were also estimated using the following equations, respectively: TSI = STV/BW × 100, in which STV is the seminiferous tubule volume and BW is the body weight [28], and ESI = SEV/BW × 100, so that SEV is the seminiferous epithelium volume.

The seminiferous tubule diameter (STD) was considered the mean of 20 random circular seminiferous tubule cross sections from each animal regardless the tubule stage because the tubular diameter does not change in adult male mice throughout the seminiferous epithelium cycle [31]. These tubule cross sections were also used to obtain the seminiferous epithelium height (SEH), which was considered as the mean of two diametrically opposed measurements made from the tunica propria to the tubular lumen. The lumen diameter was calculated subtracting the two heights of the seminiferous epithelium from STD [32].

The seminiferous tubule area (STAr) was determined using the following equation: STAr = *πR*^2^, in which *R* is the tubule radius. The lumen area (LAr) was calculated by the following equation: LAr = LR^2^, where LR is the luminal radius. The epithelium area (EAr) was obtained by subtracting STAr from LAr. The area results were expressed as square millimeter (mm^2^). The tubule epithelium ratio (TER) was obtained by dividing the STAr by the EAr [14].

The seminiferous tubule length (STL) was estimated using the following equation: STL = STV/*πR*^2^ (STV is the seminiferous tubule volume, *πR*^2^ is the tubule area, and *R* = diameter/2) [33]. Afterward, the STL values were divided by the testicular weight to obtain the seminiferous tubule length per gram of testis (STL/g).

### 2.9. Intertubular Histomorphometry Histomorphometric Evaluation of Intertubule

The proportion among the intertubular components (Leydig cell nucleus and cytoplasm, blood vessels, lymphatic space, macrophages, and connective tissue) was calculated after counting 1,000 intersection points, per animal, over the intertubular compartment. The following equation was used to calculate the percentage of such components: the percentage of each intertubular component = number of points on the component × 100/1000 (total points). The following equation was applied to calculate the volume (mL) of each component: (percentage of the element in the testes × testicular parenchyma weight)/100 [34].

The diameter of the Leydig cell nucleus was considered the mean of 30 nuclear diameters per animal (400x magnification), which had characteristic perinuclear chromatin and evident nucleoli. Nuclear (NV) and cytoplasmic volumes (CV) (NV = 4/3(*πR*^3^), *R* = nuclear diameter/2, and CV = %cytoplasm × NV/%nucleus, respectively) were calculated, followed by the calculation of the single Leydig cell volume (LV = NV + CV, in *μ*m^3^) [34].

The volume occupied by the Leydig cells in the testis was determined as follows: percentage of Leydig cells in the testicular parenchyma × parenchyma weight of the testes/100. Afterward, the volume occupied by Leydig cells in the testis was divided by the testicular weight to obtain the volume that the Leydig cells occupy per gram of testis [14].

The total number of Leydig cells in the testes was determined using the following equation: volume occupied by Leydig cells in the testicular parenchyma (*μ*m^3^)/Leydig cell individual volume (*μ*m^3^). The total number of Leydig cells per gram of testis was calculated using the following equation: volume that the Leydig cell occupies per gram of testis (*μ*m^3^)/volume of one Leydig cell (*μ*m^3^). The Leydigsomatic index (LSI) was calculated using the following equation: LSI = total volume of Leydig cell in the testicular parenchyma/BW × 100, in which BW is the body weight [32].

### 2.10. Serum Testosterone Quantification

The blood was collected during anesthesia by cardiac puncture and centrifuged at 419 × g for 15 min. The serum was stored in microtubes and frozen at -20°C. The serum testosterone was quantified by chemiluminescent assay, using the Access testosterone reagent kit, suitable for the Access 2 Immunoassay System (Beckman Coulter, Brea, CA).

### 2.11. Statistical Analysis

The percentage values were converted into sine arc to increase the statistical analysis accuracy [35]. Normal distribution was investigated by Shapiro-Wilk's test. The results obtained from the quantitative evaluations were assessed by the analysis of variance (ANOVA), followed by the Student-Newman-Keuls (SNK) post hoc method, using Statistica 7.0 program. The differences were considered significant when *p* ≤ 0.05. Data were expressed as mean ± standard deviation of the mean (SEM).

## 3. Results

### 3.1. Cadmium Bioaccumulation and Micromineral Levels

**Figure 1:**
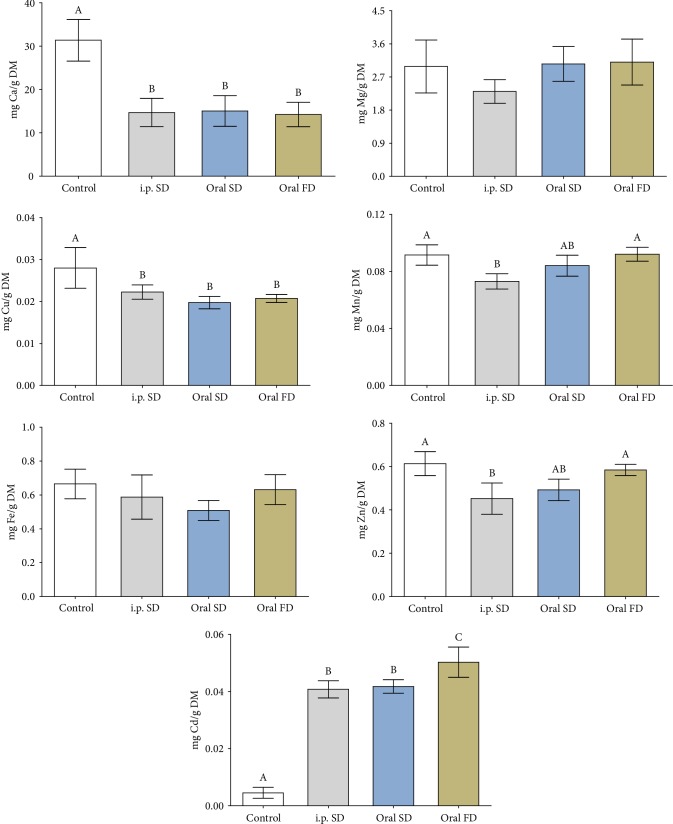
Levels of cadmium and testicular essential minerals (g/DM) of mice exposed to cadmium chloride (CdCl_2_). Control: distilled water; i.p. SD: CdCl_2_ intraperitoneal single dose; oral SD: CdCl_2_ oral single dose; oral FD: CdCl_2_ oral fractionated dose; DM: dry mass; Ca: calcium; Mg: magnesium; Cu: copper; Mn: manganese; Fe: iron; Zn: zinc; Cd: cadmium. (A, B) Different letters indicate significant differences by the Student-Newman-Keuls test (*p* ≤ 0.05).

The concentration of testicular Cd increased in all routes of administration of the metal compared to the control, mainly in the oral FD, while Ca and Cu decreased in all groups exposed to Cd (Figure 1, *p* ≤ 0.05). The concentration of manganese (Mn) and zinc (Zn) decreased in animals that received Cd *i.p.* SD in comparison to the control and oral FD groups, similar to the oral SD (Figure 1, *p* ≤ 0.05).

### 3.2. Oxidative Status Analyses

**Figure 2:**
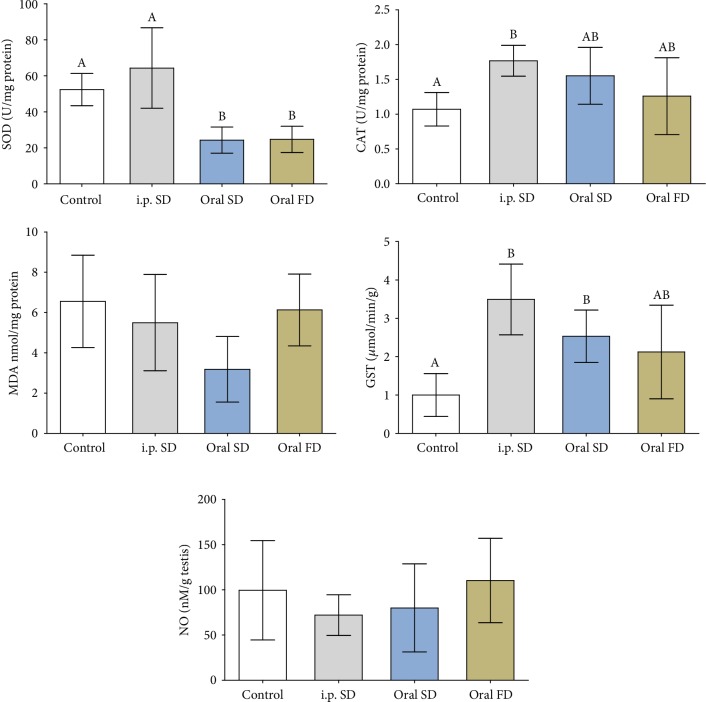
Activity of antioxidant enzymes, oxidative stress marker, and nitric oxide levels of mice exposed to cadmium chloride (CdCl_2_). Control: distilled water; i.p. SD: CdCl_2_ intraperitoneal single dose; oral SD: CdCl_2_ oral single dose; oral FD: CdCl_2_ oral fractionated dose; SOD: superoxide dismutase; CAT: catalase; GST: glutathione S-transferase; MDA: malondialdehyde; NO: nitric oxide. (A, B) Different letters indicate significant differences by the Student-Newman-Keuls test (*p* ≤ 0.05).

SOD activity decreased in the animals that received oral Cd in both routes of administration. CAT activity increased in relation to the control group only in the animals that received Cd *i.p.*, similar to the oral routes. The activity of GST increased in *i.p.* and oral SD routes in relation to the control group (Figure 2, *p* ≤ 0.05). However, the activity of this enzyme did not differ between the groups that received Cd. MDA and NO levels were not altered by Cd exposure (Figure 2, *p* > 0.05).

### 3.3. Biometric Parameters

Testicular and parenchyma weights decreased in animals that received oral Cd in both routes of administration (Table 1, *p* ≤ 0.05). There were no changes either in body weight or in gonadosomatic and parenchymosomatic indexes in all experimental groups (Table 1, *p* > 0.05).

### 3.4. Testicular Histopathology

**Figure 3:**
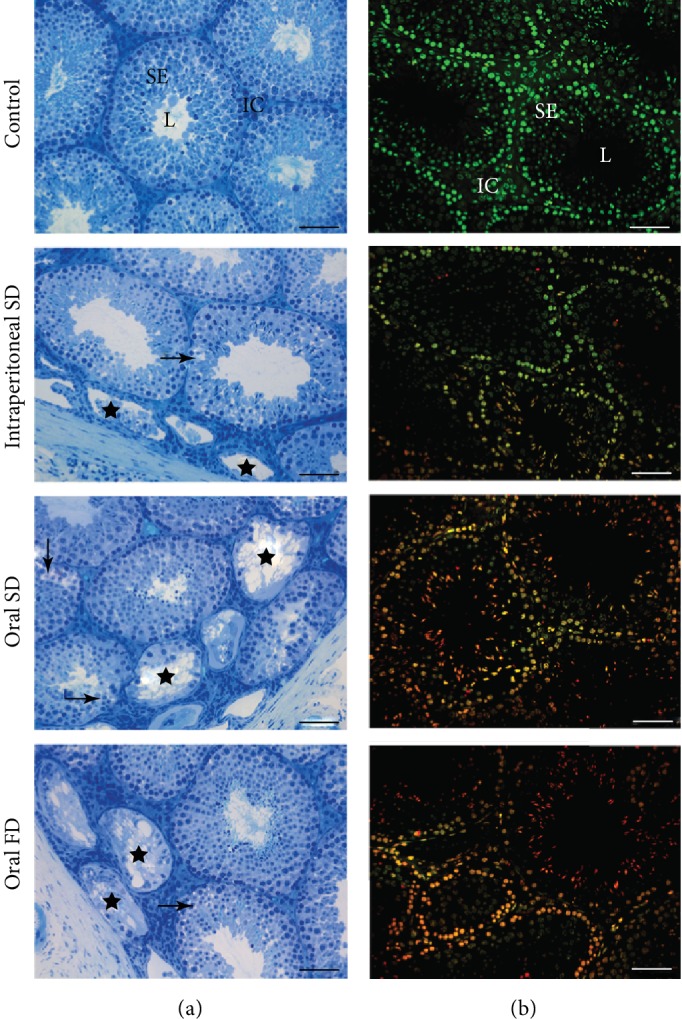
Photomicrographs of testicular sections from control and of mice exposed to cadmium chloride (CdCl_2_). On (B), sections show the tubular compartment composed of a seminiferous epithelium (SE) and lumen (L) and an intertubular compartment (IC) analyzed under light microscopy with toluidine blue. Arrows: vacuolated germinal epithelium (mild pathology); stars: degenerate seminiferous tubules (severe pathology). On (B), sections of seminiferous epithelium analyzed under epifluorescence microscopy using the fluorochrome dye acridine orange (AO; green) and propidium iodide (PI; red). Viable cells (green) and nonviable cells with initial damage (orange) and positive propidium iodide cells (red). Control: distilled water; intraperitoneal SD: CdCl_2_ intraperitoneal single dose; oral SD: CdCl_2_ oral single dose; oral FD: CdCl_2_ oral fractionated dose; SE: seminiferous epithelium; L: lumen; IC: intertubular compartment. Bars = 60 *μ*m.

**Figure 4:**
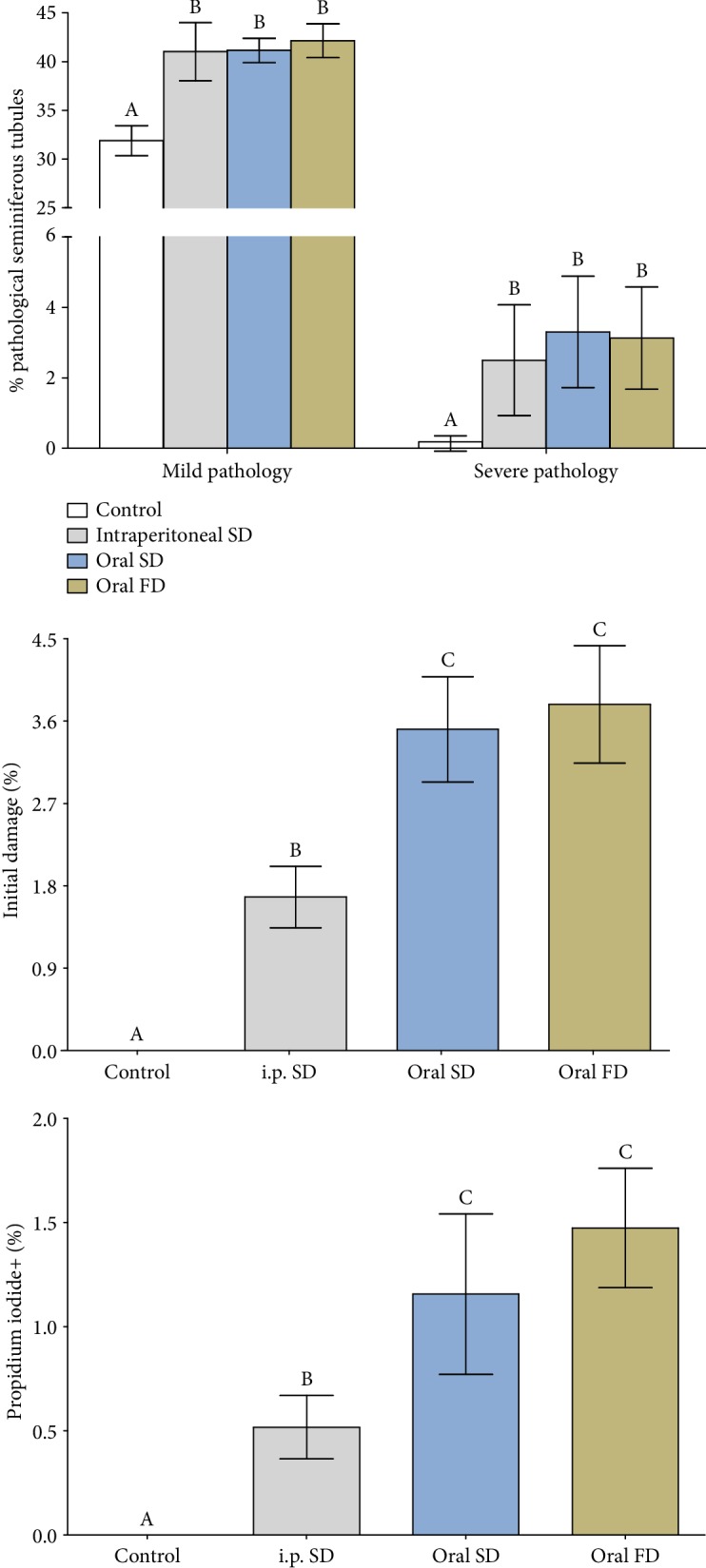
Percentage of pathological seminiferous tubules and cell damage of mice exposed to cadmium chloride (CdCl_2_). Control: distilled water; intraperitoneal SD: CdCl_2_ intraperitoneal single dose; oral SD: CdCl_2_ oral single dose; oral FD: CdCl_2_ oral fractionated dose. (A, B) Different letters indicate significant differences by the Student-Newman-Keuls test (*p* ≤ 0.05).

The changes in tissue architecture observed in animals exposed to Cd include degeneration of the seminiferous tubules with absence of germ cells and generalized vacuolization of the seminiferous epithelium (Figure 3). Therefore, the percentage of seminiferous tubules with mild and severe pathologies increased in all animals exposed to Cd (Figure 4, *p* ≤ 0.05).

### 3.5. Germ Cell Viability

Animals exposed to Cd showed increased proportions of cells with initial damage, evidenced by the overlap of acridine orange and propidium iodide (merge), and positive propidium iodide cells (Figure 3). However, this percentage was higher in animals in which Cd was administered orally in both single and fractionated doses (Figure 4, *p* ≤ 0.05).

### 3.6. Tubular Histomorphometry

The percentage and volume of the lumen increased in the animals that received Cd *i.p.* SD (Tables 2 and 3, *p* ≤ 0.05). However, the lumen volume decreased in animals treated with Cd oral SD compared to the control group but was similar to Cd oral FD (Table 3, *p* ≤ 0.05). The percentage of the epithelium decreased in animals exposed to Cd *i.p.* SD (Table 2, *p* ≤ 0.05). However, the epithelium volume was reduced only in animals exposed to Cd oral FD compared to the control group, similar to the groups exposed to Cd. The epithelium height decreased in the Cd oral FD group in relation to the control and Cd oral SD groups (Table 3, *p* ≤ 0.05).

The volume of seminiferous tubules was reduced in animals in which Cd was administered orally in both single and fractionated doses (Table 3, *p* ≤ 0.05). The tubule epithelium ratio increased in animals exposed to Cd oral FD in relation to the other experimental groups (Table 3, *p* ≤ 0.05). The other tubular morphometric parameters did not change after Cd exposure in both routes (Table 3, *p* > 0.05).

### 3.7. Intertubular Histomorphometry

The intertubular volume decreased in the animals that received Cd oral FD compared to Cd *i.p.* SD (Table 4, *p* ≤ 0.05). The percentage of connective tissue was higher in animals exposed to Cd oral SD, while their volume increased in these same animals, compared to the control and Cd oral FD groups (Table 4, *p* ≤ 0.05). The percentage of macrophages increased in all routes of Cd exposure, while the volume increased in both forms of oral administration (Table 4, *p* ≤ 0.05). The blood vessel volume decreased when Cd was offered orally SD compared to the control animals and those that received Cd *i.p.* SD (*p* ≤ 0.05), but it was similar to the oral FD group (Table 4, *p* > 0.05). The percentage of the Leydig cytoplasm increased in animals that received Cd *i.p*. in comparison to the other experimental groups (Table 4, *p* ≤ 0.05). The percentage of the Leydig nucleus was higher in the animals that received Cd orally SD when compared to the control (Table 4, *p* ≤ 0.05). The Leydig cytoplasm volume decreased when Cd was administered orally FD compared to the control and Cd *i.p.* groups (Table 4, *p* ≤ 0.05). However, the administration of Cd via *i.p.* increased the cytoplasmic volume of Leydig cells (Table 4, *p* ≤ 0.05) in relation to the other experimental groups.

There were no changes in the stereological parameters of Leydig cells, such as nuclear diameter, cytoplasmic and nuclear volumes, and cell numbers in animals exposed to Cd (Supplementary [Supplementary-material supplementary-material-1], *p* > 0.05).

### 3.8. Serum Testosterone Quantification

The concentration of serum testosterone decreased in animals exposed to oral Cd (Supplementary [Supplementary-material supplementary-material-1], *p* ≤ 0.05). Animals receiving Cd *i.p.* did not show any change in serum testosterone concentration (Supplementary [Supplementary-material supplementary-material-1], *p* > 0.05).

## 4. Discussion

Cadmium exposure led to reduction in testicular and parenchyma weights in animals receiving Cd oral SD and FD. However, body weight did not change in both evaluated routes. Cupertino et al. [6] did not observe changes in biometric parameters of rats receiving 1.2 mg/kg of CdCl_2_ in a single dose and evaluated after 7 days, as well as in mice receiving 2 mg/kg for 7 consecutive days [36], both by the *i.p.* route. On the other hand, rats receiving 1.2 mg/kg of CdCl_2_*i.p.* showed reduction in testicular weight [7]. Differently from the present study, rats receiving 50 mg/kg of CdCl_2_ by the oral route, 3 times a week for 15 days, showed no change in body and testicular biometric parameters [37]. According to the authors, this finding is indicative of nonoccurrence of general toxicity of Cd, and this may present greater toxicity when administered by the *i.p.* route. However, the results of the present study suggest that the oral route presents greater toxicity than the *i.p.* route.

The accumulation of Cd was demonstrated by the higher concentration of this metal in the testis of animals exposed to oral FD. The greater accumulation in this route may be related to the daily administration of the metal, which maintains the continuous absorption throughout the experimental period.

According to Hentze et al. [10], Cd can compete with minerals for the divalent metal transporter (DMT-1) located in the duodenum, which has specificity for Fe, Zn, and Mg, and reduce the absorption of these minerals. This transporter has been reported to be expressed in cells of the seminiferous epithelium (Sertoli and germ cells) of the adult animals, and its expression is associated with the stages of the seminiferous tubule [38]. However, in the present study, the *i.p.* route was more effective in reducing the concentration of minerals than the oral route. Therefore, the effects of this competition could not be observed in the oral administration route.

In the present study, we observed no changes in testicular Fe concentration in animals after *i.p.* and oral Cd exposure. Differently, mice receiving 25 mg/kg of CdCl_2_, once a week for 35 days, showed a reduction in testicular Fe concentration [39]. In another study, these authors also observed reduction in this mineral in animals receiving 50 mg/kg of CdCl_2_ once a week for 60 days [40]. According to the authors, the Cd exposure leads to the expulsion of testicular Fe, which leads to its decreased concentration. However, in both studies, the time of Cd exposure was higher than in the present study.

Cd has the ability to compete with Cu at the site of membrane and cytoplasmic proteins [6]. The reduced Cu concentration in both routes of administration may be related to this competition capacity. In addition, the route did not affect the testicular concentration of this mineral, since the concentration of Cu decreased in both routes of Cd exposure. It is known that Cu, together with Zn and Mn, works as a cofactor of SOD [12]. Thus, the lower concentration of this mineral may be related to the reduced SOD activity in the oral route of Cd administration, with induced oxidative stress [41]. The reduction in testicular SOD activity after Cd exposure related to increased oxidative stress has already been described [42–44]. The reduced enzyme activity may have been caused by excess of the superoxide radical in the oral route, with consequent enzymatic saturation [45] or by inactivation of enzymatic functional groups [42, 46, 47]. In addition, imbalance of essential minerals may lead to altered SOD expression [48]. However, although they are SOD cofactors, Zn and Mn reduction in animals receiving Cd *i.p.* SD did not result in loss of enzymatic activity.

Increased CAT activity in *i.p.* SD also indicates the occurrence of oxidative stress due to the higher production of H_2_O_2_ by SOD in this route, to be decomposed through CAT. In addition, according to Cupertino et al. [6], increased CAT activity in animals exposed to Cd may be a demonstration of the attempt to protect the tissue against changes caused by the metal.

GST belongs to the second antioxidant defense line, whose function is the detoxification of xenobiotics, with consequent protection against the oxidative stress [49], besides the detoxification of peroxidized lipids [45]. The increase in testicular MDA concentration associated with reduction of GSH [36, 50, 51] and GST [12], after Cd exposure, is indicative of lipid peroxidation and oxidative stress. As in the present study, the MDA concentration did not change; the treatment may not have promoted lipid peroxidation. The increased GST shows that this enzyme activity was not impaired by Cd.

Chemical similarities between Cd and Ca atoms make it possible for Cd to replace Ca in binding proteins [52]. Thus, the reduced concentration of testicular Ca found in both administration routes may be related to its replacement by Cd. Thus, the replacement of Ca in the cellular junctions can lead to the destabilization and consequent rupture of the blood-testis barrier [53]. Previous studies have shown that mice exposed to Cd presented a decreased number of proteins that form the blood-testis barrier, such as cadherins, catenins, occludin, and claudin-11 [8, 54]. Thus, changes in the proteins present in the barrier can be harmful to the germinal epithelium [9]. Therefore, the higher percentage of tubular pathologies found in animals exposed to Cd, in both administration routes, may be related to the low concentration of Ca and the bioaccumulation of Cd.

Changes in morphometric parameters, such as reduced tubule and epithelial volumes and decreased epithelial height, associated with increased tubule epithelium ratio (TER) in animals receiving Cd oral route fractions, may indicate loss of the germinative epithelium [6, 7]. Such loss may be due to the destabilization of the blood-testis barrier caused by the reduced Ca concentration [8, 9]. According to França and Russell [31], the loss of germ cells leads to changes in morphometry and decline in quantitative parameters, as observed in the present study. Thus, it is suggested that the reduced height of the epithelium, associated with increased TER, may lead to spermatogenic capacity loss. The reduced volume of the seminiferous tubule in the oral route, besides being related to the lower testicle weight of these animals, may also be associated with the smaller epithelium volume in the oral FD route, which contributed to the reduction of epithelial height. The higher percentage of positive propidium iodide cells, especially in the oral route, may also be related to loss of germ cell viability and consequent loss of the epithelium [27].

Considering the oral FD route, the greater intensity of changes in tubular morphometry may be related to the higher Cd accumulation and not to oxidative stress. Dodson et al. [55] challenge the paradigm that oxidative stress and ROS generation are the main causes of pathologies. The authors showed that low doses of arsenic (As) cause proteostasis and not oxidative stress in cell culture. In addition, Wang et al. [56] reported that excessive Cd exposure can lead to cellular changes due to Cd accumulation. According to the authors, the cells perform autophagy as a form of defense. However, autophagy is intensified when there is metal accumulation, and tissue damage can be observed due to the large amount of cells in the process of cell death [56]. Thus, the greatest tubular damage found in the oral FD route may be related to increased Cd accumulation in the testis of these animals, with consequent loss of the germinal epithelial cells due to increased autophagy and destabilization of the blood-testis barrier. However, further studies should be performed to confirm this process.

The results found by Sharma and Kaur [57] corroborate the findings of the present study. The authors observed that mice exposed to small doses (0.1 mg/kg) of CdCl_2_*i.p*. daily, for 15 and 30 days, showed greater alteration in testicular histology compared to those exposed to 2 mg/kg *i.p.* in a single dose and evaluated after 15 and 30 days of exposure. Cupertino et al. [6] reported vacuolization of germ cells and reduced tubule and epithelial volumes in rats receiving 1.2, 1.4, 1.8, and 2.2 mg/kg of CdCl_2_, in a single dose *i.p.*, in addition to tubular obstruction in those receiving at least 1.4 mg/kg. These same authors observed increased testicular Cd concentration in all animals exposed to the metal. However, unlike the present study, the authors reported higher germinative epithelium loss, especially in animals that received 1.8 and 2.2 mg/kg of CdCl_2_, to increased testicular concentration of Ca and consequent dystrophic calcification. Predes et al. [4, 58] also observed disorganized seminiferous tubules, besides reduced testicular weight of Wistar rats receiving a single dose of 1.2 mg/kg CdCl_2_*i.p.* and evaluated after 7 days. The authors observed reduced tubular diameter after 56 days of metal exposure, which gives evidence of the progression of tubular alterations caused by Cd, and spermatogenesis impairment.

The increased percentage and volume of the lumen associated with reduced percentage of the epithelium observed in animals that received Cd *i.p.* did not lead to changes in the other tubular parameters. Since proportion is a spot analysis and the values obtained are used to determine the global parameters of the organ, it is concluded that the *i.p.* route did not cause the same intensity of toxicity shown by the oral route in the tubular parameters.

The increased percentage of connective tissue was significant only in animals that received Cd oral SD, although its proportion was also higher in other Cd-administrated groups. It is known that the presence of macrophages induces the synthesis of collagen via fibroblasts [59]. Thus, the greater proportion of connective tissue may be associated with an increased percentage of macrophages in animals exposed to Cd. In addition, the excess fibers can impair the communication between tubular and intertubular compartments and damage the spermatogenesis. Testicular fibrosis caused by Cd exposure is already known [60], as well as the increased percentage of macrophages [61].

The increased percentage of the Leydig cell nucleus in the oral SD route, along with the increased cytoplasmic percentage and cytoplasmic and cell volumes in *i.p.* SD administration, may be due to the onset of the necrotic cell death process. According to Majno and Joris [62], cells in necrosis show increased volume due to the destabilization of the plasma membrane, followed by rupture and cell death. Although no decrease was observed in the number of Leydig cells, in the oral route, the serum concentration of testosterone decreased. Thus, the reduction in serum testosterone concentration may be due to morphometric changes in Leydig cells, which suggests the steroidogenic impairment of this route, compared to the *i.p.* route. Such loss may have increased the intensity of the tubular changes found in the oral route.

The oral FD route presented greater intensity of testicular morphometric changes and lower oxidative stress. The lower oxidative stress may have occurred due to the adaptation to ROS generation caused by continuous exposure to Cd. This adaptive mechanism is reported in patients with *β*-thalassemia in order to maintain homeostasis due to Fe overload [63], in pancreatic *β*-cells after As exposure [64], and in zebrafish after fluoroquinolone exposure [65]. Thus, it is suggested that the testis also has an oxidative adaptive mechanism in case of continuous exposure to Cd. However, the possible adaptation to stress conditions was not effective in tissue protection. Thus, we believe that continuous and low-dose exposure of Cd causes serious damage on the testis compared to single-dose exposure of this metal.

## 5. Conclusion

The subacute exposure leads to the bioaccumulation of this metal, regardless of the route of administration, although it is more significant in the oral fractionated route. The route of administration does not affect the reduction of testicular Ca and Cu, whereas the *i.p.* route reduces Mn and Zn concentration. SOD activity decreases only when the metal is administered orally. Changes in the tubular parameters and cell viability are observed in both routes, but it is more intense in the oral route, mainly when fractionated. Serum testosterone concentration decreases when Cd is given orally. The lower serum testosterone concentration may be related to the decreased testicular and parenchyma weights and increased intensity of morphological damage in the testis of animals receiving oral Cd, mainly in the fractioned dose. Thus, the oral administration of Cd showed greater testicular damage potential, mainly when the metal is administered in a fractionated dose.

## Figures and Tables

**Table 1:** Body and testicular biometry of mice exposed to cadmium chloride (CdCl_2_) (*n* = 5 animals/group).

	Control	i.p. SD	Oral SD	Oral FD
Body weight (g)	38.10 ± 0.47	39.60 ± 4.00	33.71 ± 4.30	36.57 ± 4.20
Testicular weight (g)	0.220 ± 0.020ª	0.216 ± 0.017ª	0.188 ± 0.016^b^	0.181 ± 0.013^b^
Parenchyma weight (g)	0.202 ± 0.017ª	0.206 ± 0.022^a^	0.175 ± 0.021^b^	0.171 ± 0.011^b^
Albuginea weight (g)	0.018 ± 0.009	0.010 ± 0.005	0.013 ± 0.007	0.010 ± 0.004
GSI (%)	0.58 ± 0.06	0.55 ± 0.02	0.57 ± 0.11	0.50 ± 0.07
PSI (%)	0.53 ± 0.05	0.52 ± 0.02	0.53 ± 0.11	0.47 ± 0.07

Mean ± SDM (standard deviation of the mean); control: distilled water; i.p. SD: CdCl_2_ intraperitoneal single dose; oral SD: CdCl_2_ oral single dose; oral FD: CdCl_2_ oral fractionated dose; GSI: gonadosomatic index; PSI: parenchymosomatic index. ^ab^Different letters in the same row, for each evaluated time, indicate significant differences by the Student-Newman-Keuls test (*p* ≤ 0.05).

**Table 2:** Volumetric density of the tubular compartment of mice exposed to cadmium chloride (CdCl_2_) (*n* = 5 animals/group).

	Control	i.p. SD	Oral SD	Oral FD
Tubule (%)	88.80 ± 0.41	87.75 ± 1.52	88.12 ± 1.10	88.74 ± 1.07
Epithelium (%)	64.72 ± 1.21ª	61.12 ± 1.72^b^	65.32 ± 1.65ª	63.98 ± 2.14ª
Tunica propria (%)	10.75 ± 0.22	10.38 ± 0.84	10.49 ± 0.60	10.65 ± 0.71
Lumen (%)	13.33 ± 1.05ª	16.25 ± 1.09^b^	12.31 ± 1.91ª	14.11 ± 0.94ª
TSI (%)	0.47 ± 0.05	0.46 ± 0.02	0.47 ± 0.09	0.42 ± 0.06
ESI (%)	0.34 ± 0.03	0.32 ± 0.02	0.35 ± 0.07	0.30 ± 0.04

Mean ± SDM (standard deviation of the mean); control: distilled water; i.p. SD: CdCl_2_ intraperitoneal single dose; oral SD: CdCl_2_ oral single dose; oral FD: CdCl_2_ oral fractionated dose; TSI: tubulesomatic index; ESI: epithelium somatic index. ^ab^Different letters in the same row, for each evaluated time, indicate significant differences by the Student-Newman-Keuls test (*p* ≤ 0.05).

**Table 3:** Morphometry and stereology of tubular compartment of mice exposed to cadmium chloride (CdCl_2_) (*n* = 5 animals/group).

	Control	i.p. SD	Oral SD	Oral FD
*Volume (mL)*				
Tubule	0.179 ± 0.015ª	0.181 ± 0.021ª	0.154 ± 0.016^b^	0.151 ± 0.010^b^
Epithelium	0.130 ± 0.010ª	0.126 ± 0.014^ab^	0.115 ± 0.014^ab^	0.109 ± 0.008^b^
Tunica propria	0.022 ± 0.002	0.021 ± 0.003	0.018 ± 0.003	0.018 ± 0.002
Lumen	0.027 ± 0.004ª	0.034 ± 0.005^b^	0.021 ± 0.001^c^	0.024 ± 0.002^ac^
Tubular diameter (*μ*m)	225.11 ± 9.04	222.41 ± 11.36	226.81 ± 11.0	215.27 ± 14.86
Lumen diameter (*μ*m)	74.19 ± 3.89	75.42 ± 6.39	80.21 ± 5.31	83.23 ± 7.70
Epithelium height (*μ*m)	150.92 ± 6.86ª	146.99 ± 11.10ª^b^	146.60 ± 5.88^a^	132.04 ± 10.77^b^
STAr (mm^2^)	0.0398 ± 0.0032	0.0389 ± 0.0040	0.0404 ± 0.0039	0.0365 ± 0.0050
LAr (mm^2^)	0.0043 ± 0.0004	0.0045 ± 0.0007	0.0051 ± 0.0007	0.0053 ± 0.0010
EAr (mm^2^)	0.0355 ± 0.0029	0.0344 ± 0.0038	0.0354 ± 0.0033	0.0311 ± 0.0044
TER	1.12 ± 0.01ª	1.13 ± 0.03ª	1.14 ± 0.01^a^	1.17 ± 0.03^b^
STL (m)	4.50 ± 0.31	4.71 ± 0.85	3.83 ± 0.38	4.20 ± 0.58
STL/g (m/g)	20.58 ± 2.28	21.68 ± 2.60	20.42 ± 2.23	23.24 ± 3.07

Mean ± SDM (standard deviation of the mean); control: distilled water; i.p. SD: CdCl_2_ intraperitoneal single dose; oral SD: CdCl_2_ oral single dose; oral FD: CdCl_2_ oral fractionated dose; STAr: seminiferous tubule area; LAr: lumen area; EAr: epithelium area; TER: tubule epithelium ratio; STL: seminiferous tubule length; STL/g: seminiferous tubule length per gram of testis. ^ab^Different letters in the same row, for each evaluated time, indicate significant differences by the Student-Newman-Keuls test (*p* ≤ 0.05).

**Table 4:** Volumetric density and volume of intertubular compartment in the testes of mice exposed to cadmium chloride (CdCl_2_) (*n* = 5 animals/group).

	Control	i.p. SD	Oral SD	Oral FD
*Volumetric density (%)*				
Intertubule	11.20 ± 0.41	12.25 ± 1.50	11.88 ± 1.12	11.26 ± 1.07
Connective tissue	0.70 ± 0.19ª	0.92 ± 0.17ª	1.36 ± 0.25^b^	0.94 ± 0.17ª
Lymphatic space	0.92 ± 0.43	0.42 ± 0.24	0.56 ± 0.11	0.87 ± 0.51
Blood vessel	1.92 ± 0.48	1.79 ± 0.42	1.22 ± 0.41	1.51 ± 0.42
Macrophage	0.23 ± 0.02ª	0.50 ± 0.21^b^	0.78 ± 0.20^b^	0.60 ± 0.19^b^
Leydig cytoplasm	6.09 ± 0.53^ab^	7.06 ± 1.05^b^	6.22 ± 0.37ª	5.84 ± 0.23ª
Leydig nucleus	1.33 ± 0.25ª	1.56 ± 0.25^ab^	1.74 ± 0.18^b^	1.50 ± 0.18^ab^
Leydig cell	7.42 ± 0.75	8.62 ± 1.30	7.96 ± 0.54	7.34 ± 0.35
*Volume (mL)*				
Intertubule	0.023 ± 0.001^ab^	0.025 ± 0.002ª	0.022 ± 0.005^ab^	0.019 ± 0.002^b^
Connective tissue	0.0014 ± 0.0004ª	0.0019 ± 0.0003ª^b^	0.0024 ± 0.0006^b^	0.0016 ± 0.0003ª
Lymphatic space	0.0019 ± 0.0009	0.0009 ± 0.0005	0.0010 ± 0.0003	0.0015 ± 0.0009
Blood vessel	0.0038 ± 0.0008ª	0.0037 ± 0.0009ª	0.0022 ± 0.0010^b^	0.0026 ± 0.0008ª^b^
Macrophage	0.0005 ± 0.0001ª	0.0010 ± 0.0005^ab^	0.0014 ± 0.0004^b^	0.0010 ± 0.0003^b^
Leydig cytoplasm	0.0123 ± 0.0014ª	0.0144 ± 0.0014^b^	0.0110 ± 0.0018^ac^	0.099 ± 0.0005^c^
Leydig nucleus	0.0027 ± 0.0004	0.0032 ± 0.0004	0.0031 ± 0.0006	0.0025 ± 0.0003
Leydig cell	0.0150 ± 0.0016ª	0.0176 ± 0.0017^b^	0.0141 ± 0.0024^a^	0.0124 ± 0.0007^a^

Mean ± SDM (standard deviation of the mean); control: distilled water; i.p. SD = CdCl_2_ intraperitoneal single dose; oral SD: CdCl_2_ oral single dose; oral FD: CdCl_2_ oral fractionated dose. ^ab^Different letters in the same row, for each evaluated time, indicate significant differences by the Student-Newman-Keuls test (*p* ≤ 0.05).

## Data Availability

The data used to support the findings of this study are available from the corresponding author upon request.

## Supplementary Materials

Supplementary Materials Supplementary Table 1: Leydig cell morphometry of mice exposed to cadmium chloride (CdCl2) (*n* = 5 animals/group).
