# Supplementary material for: Subacute Testicular Toxicity to Cadmium Exposure Intraperitoneally and Orally
Source: Oxid Med Cell Longev. 2019 Nov 25;2019:3429635. doi: 10.1155/2019/3429635 (PMC6899267; doi:10.1155/2019/3429635)
Supplement: Supplementary Materials — Supplementary Table 1: Leydig cell morphometry of mice exposed to cadmium chloride (CdCl2) (n = 5 animals/group). [file 3429635.f1.pdf]

Supplementary Table 1. Leydig cell morphometry of mice exposed to cadmium chloride ($CdCl_2$) (n = 5 animals/group).

	Control	I.p. SD	Oral SD	Oral FD
Nuclear diameter (μm)	5.01 ± 0.23	4.98 ± 0.25	5.21 ± 0.30	5.09 ± 0.15
LSI (%)	0.039 ± 0.004	0.045 ± 0.007	0.043 ± 0.011	0.034 ± 0.004
Nuclear volume (μm^3)	66.37 ± 9.43	64.88 ± 9.42	74.87 ± 13.16	69.15 ± 5.88
Cytoplasmic volume (μm^3)	316.06 ± 92.76	294.72 ± 40.04	268.46 ± 43.01	273.92 ± 51.76
Cell volume (μm^3)	382.43 ± 102.13	359.60 ± 49.31	343.33 ± 55.66	343.07 ± 57.06
Leydig volume/g testis (mL)	0.068 ± 0.008	0.082 ± 0.011	0.074 ± 0.008	0.069 ± 0.004
Number of cells ($\times 10^6$)	41.17 ± 9.43	49.28 ± 4.91	41.01 ± 4.38	37.29 ± 7.31
Number of cells/g testis ($\times 10^6$)	191.10 ± 52.89	227.82 ± 10.13	215.50 ± 33.39	205.98 ± 35.80
Testosterone (ng/dL)	946.21 59.64[a]	942.84 242.98[a]	64.26 5.82[b]	53.21 17.79[b]

Mean ± SDM (standard deviation of the mean); Control = Distilled Water; I.p. SD = $CdCl_2$ Intraperitoneal Single Dose; Oral SD = $CdCl_2$ Oral Single Dose; Oral FD = $CdCl_2$ Oral Fractionated Dose. LSI = Leydigosomatic index. [ab]Different letters in the same row, for each evaluated time, indicate significant differences by Student test Newman Keuls ($p \leq 0.05$).
